# Left Preference for Sport Tasks Does Not Necessarily Indicate Left-Handedness: Sport-Specific Lateral Preferences, Relationship with Handedness and Implications for Laterality Research in Behavioural Sciences

**DOI:** 10.1371/journal.pone.0105800

**Published:** 2014-08-20

**Authors:** Florian Loffing, Florian Sölter, Norbert Hagemann

**Affiliations:** 1 Institute of Sports and Sports Science, University of Kassel, Kassel, Germany; 2 Institute of Sport and Exercise Sciences, University of Muenster, Muenster, Germany; University of Utah, United States of America

## Abstract

In the elite domain of interactive sports, athletes who demonstrate a left preference (e.g., holding a weapon with the left hand in fencing or boxing in a ‘southpaw’ stance) seem overrepresented. Such excess indicates a performance advantage and was also interpreted as evidence in favour of frequency-dependent selection mechanisms to explain the maintenance of left-handedness in humans. To test for an overrepresentation, the incidence of athletes' lateral preferences is typically compared with an expected ratio of left- to right-handedness in the normal population. However, the normal population reference values did not always relate to the sport-specific tasks of interest, which may limit the validity of reports of an excess of ‘left-oriented’ athletes. Here we sought to determine lateral preferences for various sport-specific tasks (e.g., baseball batting, boxing) in the normal population and to examine the relationship between these preferences and handedness. To this end, we asked 903 participants to indicate their lateral preferences for sport-specific and common tasks using a paper-based questionnaire. Lateral preferences varied considerably across the different sport tasks and we found high variation in the relationship between those preferences and handedness. In contrast to unimanual tasks (e.g., fencing or throwing), for bimanually controlled actions such as baseball batting, shooting in ice hockey or boxing the incidence of left preferences was considerably higher than expected from the proportion of left-handedness in the normal population and the relationship with handedness was relatively low. We conclude that (i) task-specific reference values are mandatory for reliably testing for an excess of athletes with a left preference, (ii) the term ‘handedness’ should be more cautiously used within the context of sport-related laterality research and (iii) observation of lateral preferences in sports may be of limited suitability for the verification of evolutionary theories of handedness.

## Introduction

Lateral preference describes humans or ‘non-human animals’ predominant use of either side of the body for carrying out specific actions. Handedness is the most prominent trait where such preference occurs [1]. While the proportion of human left-handedness varies by geographic region [2], [3] and is higher in males compared to females [4], [5], overall left-handedness is considerably lower compared to right-handedness in any culture studied so far and such asymmetry seems relatively stable since thousands of years [6], [7]. The question of which mechanisms constitute the maintenance of an imbalanced human handedness polymorphism is controversially discussed [8]–[10]. One suggestion is that left-handedness is associated with both fitness costs (e.g., higher proneness to health-related problems), resulting in frequencies considerably below 50%, and benefits, compensating for some costs and thus preserving left-handedness [11]. From an evolutionary perspective, the fighting hypothesis argues that the costs (potentially) inherent to left-handedness may be offset by negative frequency-dependent selection mechanisms [12]. More specifically, left-handers are assumed to enjoy an advantage in duel-like confrontations because of their relative rarity which, as a result, makes their opponents become less familiar with the left-handers' fighting behaviour. Such fighting advantage in male-male competition, in turn, is thought to have helped left-handers to ensure reproductive success (for a recent critique, e.g. see [13]).

Among other things, elite sporting competition databases have been used to verify the fighting hypothesis [11]. Consistent with its predictions, in professional rankings of interactive sports such as table tennis or fencing, where athletes can directly influence and constrain each other's actions, athletes who use their left hand for tasks like holding a racket or a foil were found overrepresented [12], [14], [15]. In contrast, no excess of left-handed performers has ever been reported in any non-interactive sport (e.g., darts, snooker, golf) [12], [16]. The clear division of sports where left-handers are overrepresented or not suggests that the performance demands inherent to interactive situations (e.g., anticipation of an opponent's action intentions or fast decision-making [17]–[21]) in combination with frequency-dependent mechanisms favour left-handed performers. In contrast, alternative explanations which propose that mechanisms associated with left-handedness *per se* constitute a left-handers' advantage (e.g., higher aggression [22], less lateralized motor skills [23] or more efficient neural processing [24] in left-handers) are little convincing so far (for a critical review, e.g. see [14]).

The informative value of research on handedness distribution in sports may be partly limited because potentially inappropriate normal population reference values were used. More specifically, in order to determine whether or not there is an excess of left-handed athletes in a sport, observed handedness frequencies are compared with expected frequencies thought to occur in the normal population (e.g., 10–13% left-handers; [12]). However, instead of using reference values that are related to the sport-specific task of interest, estimates of handedness distribution in the normal population were often taken from large surveys [4], [5] which reported lateral preferences for unrelated tasks like writing or throwing (for exceptions, e.g. see [16], [25], [26]). We suppose that reliance on task-*un*related comparative values is questionable, especially if these values are considerably different to the normal population's lateral preferences for the particular sport task of interest. Then, the impact of laterality on sports performance may be over- or underestimated.

Remarkably, the literature almost lacks a comprehensive database of reference values for sport-specific lateral preferences (for an exception see [27]). The above mentioned procedure seems justified either if tasks are very similar [12] or if there is evidence of a close relationship between different items [28], [29], as has been reported for throwing a ball and holding a racket [30]. Even in the absence of these two preconditions, as long as unimanually controlled sport tasks are considered (e.g., holding a weapon in fencing), comparative values derived from unrelated but clearly unilateral tasks like writing or throwing may be acceptable.

However, reliance on reference values for unrelated unimanually controlled tasks (e.g., writing or throwing) may be problematic when sport tasks are examined that are not under distinct unilateral control. Examples of such sport tasks are batting in baseball [26] or cricket [31], holding a stick in ice hockey [32], fighting stance in boxing [12] or in related combat sports such as Mixed Martial Arts (MMA) [33], [34]. For these tasks, differentiation of athletes into left- vs. right-handers is difficult given that both hands are involved in the control of motor actions (e.g., batting in baseball) or may be used during fights (e.g., in boxing). Considering evidence which suggests that lateral preferences for unilateral and bilateral tasks are not perfectly correlated (e.g., throwing a ball and batting; [26], [31]), we expect that task-specific reference values are mandatory particularly when examining bimanually controlled actions.

Moreover, up to now there is very limited understanding of the (strength of) relationship between those preferences and handedness [27], [34], [35]. Furthering our knowledge about such relationship is important as the detection of inconsistent relationships in particular may have several significant consequences for various domains of laterality research. First, inconsistent relationships would suggest that lateral preferences observed in sports may not allow reliable inferences in support of the fighting hypothesis or other evolutionary models to explain the maintenance of the polymorphism in human handedness. Second, inconsistent relationships between sport-specific lateral preferences and handedness would also question the validity of explanations such as that an excess of left preferences among elite athletes is due to handedness-dependent differences in neurological functioning. Finally, depending on the strength of relationship, it might be advisable that researchers be more cautious with usage of the term ‘handedness’ when referring to lateral preferences in sports. Such advice could be particularly relevant when dealing with sport tasks for which relationships with handedness are low.

In light of the above issues, the aim of this study was as follows. First, we sought to establish reference values for sport-specific lateral preferences in the normal population. To this end, we designed a paper-based questionnaire which comprised items on 16 different sport tasks that are executed with the hands, either unilaterally (e.g., fencing) or bilaterally (e.g., batting in baseball), the feet (e.g., kicking) or tasks that may be executed in different directions (e.g., rotating left or right when performing a pirouette in figure skating). Albeit we will report the results on all items, our focus is on uni- and bimanual sport tasks. Second, we aimed to identify the relationships between sport-specific lateral preferences and handedness. Therefore, we additionally included an established questionnaire on handedness preferences [36]. To anticipate, we will show that the frequencies of sport-specific left preferences as well as the relationship between lateral preferences and handedness vary considerably across different sport tasks.

## Material and Methods

### Ethics Statement

The study was approved by the ethics committee at the Department of Social Sciences, University of Kassel (Germany), and was performed in accordance with the ethical standards set out in the Revised Declaration of Helsinki as of November 2008. In the study proposal, the ethics committee was informed that, because our questionnaire did not contain any dubious questions or answers, legal age of participants was not required.

All participants were given written information about the study and they signed written informed consent prior to the completion of the questionnaire. Written information was related to the confidentiality of data, the ensured anonymity of data collection and analysis, the subject's right to withdraw from the study at any stage without any (negative or positive) consequences for the subject and the absence of any potential risks or benefits associated with participation in the study. Data was anonymized upon collection and the authors did not have access to identifying information.

Our sample included five minors (two males, 16 and 17 years old; three females, one 13 and two 17 years old). All participants, including the five minors, were able to comprehend the information provided prior to as well as while filling out the questionnaire. We obtained written informed consent from the minors prior to their participation. However, we did not obtain additional informed consent (neither written nor verbal) from these participants' next of kin, caretakers, or guardians because our research was clearly not suspected to create any distress or harm and only anonymous questionnaires were used. As we understand, this procedure was also in line with the current ethical principles set out by the American Psychological Association (standard 8.05).

### Participants

The sample comprised 903 randomly selected individuals (males: *n* = 408, age: 16–77 years, *M* = 25.01 years, *SD*  = 9.11; females: *n* = 495, age: 13–75 years, *M* = 22.84 years, *SD*  = 6.32) who were predominantly university students from the University of Kassel (Germany).

### Questionnaire

The 16 questions related to sport-specific tasks are summarized in Table 1. For each item, participants were asked to choose their lateral preference out of three alternatives (provided in the following order): ‘left’, ‘no preference’ or ‘right’ (German terms: ‘links’, ‘keine Bevorzugung’, ‘rechts’). To improve the participants' understanding of the meaning of left vs. right for the respective tasks, each sport-specific question was accompanied by monochrome pictures of a person performing the respective tasks in a left and right orientation (see Table S1). The individual shown on the pictures in Table S1 has given written informed consent (as outlined in PLOS consent form) to publish these images.

**Table 1:** Questions on sport-specific tasks (see also Table S1).

Sport	Task/Item	Question
e.g., darts, team-handball	throwing	Which hand would you use for throwing darts or a ball (e.g., in team-handball)?
fencing	holding a weapon	In which hand would you hold a weapon in fencing?
racket sports	holding a racket	In which hand would you hold a racket (e.g., table tennis, tennis or badminton)?
ninepin, tenpin bowling	bowling	Which hand would you use for bowling (e.g., in ninepin or tenpin)?
billiards	holding a cue	Which hand (rear hand) would you use to hold a cue to play a ball in billiards?
baseball	batting	How would you hold a baseball bat (i.e., which hand is at the top)?
ice hockey	holding a stick	How would you hold a hockey stick (i.e., which hand is at the bottom)?
boxing	stance	Which stance would you choose in boxing to fight an opponent?
golf	holding a golf club	How would you hold a golf club (i.e., which hand is at the bottom)?
target shooting	shooting orientation	Which orientation would you choose for target shooting?
archery	shooting orientation	Which orientation would you choose for doing archery?
soccer	kicking	Which foot would you use for kicking a ball?
long jump	jump off	Which leg would you use to jump off in long jump?
high jump	approaching side *	From which side would you approach the crossbar in high jump?
skateboarding, snowboarding	front foot	Which foot would you stand in front with on a skateboard or snowboard?
e.g., figure skating	rotation direction	Which direction would you prefer when rotating along the vertical axis (e.g., when doing a pirouette in figure skating)?

* *Note:* In high jump, approaching from the left means that an athlete jumps off with his or her right foot (and *vice versa*). All participants were informed in writing about this relationship when filling out the questionnaire. To facilitate understanding of lateral preference for high jump, we will report the preferred foot instead of approach side for this task.

In order to examine the relationship between sport-specific lateral preferences and handedness, the questionnaire also included a German version of the Edinburgh Handedness Inventory (EHI) [36]. The EHI comprised ten items such as writing, throwing or holding a spoon. Unlike in the original version of the EHI we provided participants with Likert-like five response alternatives, ordered as follows, of which they had to check one for each item and each alternative was assigned a different score for later analysis (scores were not available to participants): ‘exclusively left’ (−10), ‘rather left’ (−5), ‘no preference’ (0), ‘rather right’ (+5), or ‘exclusively right’ (+10) (German terms: ‘ausschlieβlich links’, ‘eher links’, ‘keine Bevorzugung’, ‘eher rechts’, ‘ausschlieβlich rechts’) [37]. We used these response modes because there is indication that participants do not strictly follow the instructions of placing one or two crosses in a ‘left’ and/or ‘right’ column as provided in the initial version of the EHI (e.g., participants just tick a box without differentiating their strength of preference by making one or two crosses) [38]. For each participant, a laterality score (LS) was calculated by summing up the ten items' scores and thus LS ranged from −100 to +100. We calculated LS instead of laterality quotients initially proposed by Oldfield [36], because laterality quotients are only sensitive to the direction of handedness, whereas the LS “is sensitive to both the degree and direction of handedness” (p. 169, [37]; see also for exemplary calculations).

Our questionnaire additionally included a German version of Coren's Lateral Preference Inventory [39], which measures hand, foot, eye and ear preference, as well as questions on the participants' current or past sporting activities. To keep focussed on our study's aim the remaining data will be reported elsewhere.

## Results

Overall, differentiation of participants into ‘left-handers’ (if LS <0) and ‘right-handers’ (if LS ≥0) revealed that our sample comprised 8.86% left-handers and that left-handers were more common in males (10.05%) compared to females (7.88%); however, the sex-dependent difference was not significant, χ^2^(1, *N* = 903)  = 1.31, *p* = .25, ω = 0.04.

**Figure 1:**
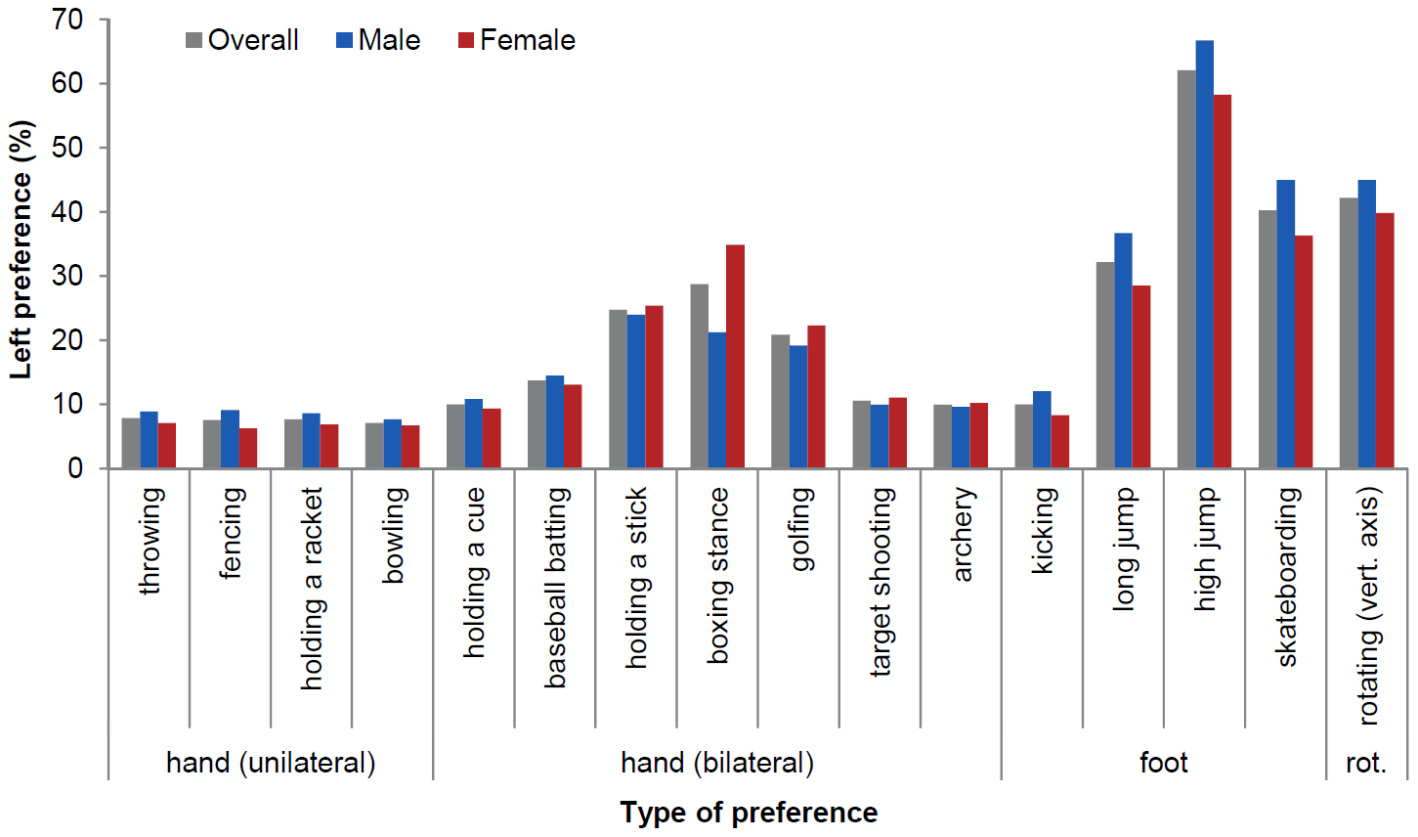
Left preferences for sport-specific tasks overall and differentiated by sex.

Lateral preferences varied considerably across the different sport-specific tasks and left preferences were higher in males compared to females in 11 out of the 16 tasks (Figure 1; raw data can be accessed in Table S2). To avoid an overestimation of left preferences, we considered responses to all alternatives (i.e., ‘left’, ‘right’ and ‘no preference’) for the calculation of left preference percentages. For tasks that require unilateral control of the hands, left preferences were quite stable, ranging from 7.62% (bowling) to 9.09% (holding a weapon) in males and from 6.26% (holding a weapon) to 7.07% (throwing) in females. However, for tasks requiring bilateral control of the hands, left preferences were clearly higher compared to the former tasks. Here, left preferences ranged from 9.61% (archery) to 23.95% (holding a stick in ice hockey) in males and from 9.31% (holding a cue in billiards) to 34.83% (boxing stance) in females. Similarly, preferences for using the left foot substantially differed as a function of task: 12.07% (kicking) to 66.67% (high jump) in males and 8.28% (kicking) to 58.25% (high jump) in females. With regard to rotating along the vertical axis, 44.94% of male and 39.84% of female participants indicated a left preference.

To determine the relationship between sport-specific lateral preferences and handedness (as represented by LS values obtained from the EHI) we calculated point-biserial correlation coefficients (*r*_pb_) for the whole sample and separately for males and females. As is illustrated in Table 2, almost any relationship was statistically significant (*p*<.05); except for rotating along the vertical axis (e.g., in figure skating) overall and in males as well as for skateboarding in males. For all unilateral manual tasks there was very good agreement between sport-specific lateral preferences and handedness. However, the relationship between handedness and tasks that require bimanual control was highly variable depending on the task and relatively low particularly for baseball batting, holding a stick in ice hockey, fighting stance in boxing and golfing. Kicking foot was moderately related to handedness. The lowest relationships were revealed for tasks where an athlete's hands are not primarily involved (i.e., long jump, high jump and skateboarding).

**Table 2:** Relationship between sport-specific lateral preferences and handedness.

		Overall		Male		Female	
Type of preference	Task	N	*r*_pb_	N	*r*_pb_	N	*r*_pb_
hand (unilateral)	throwing	888	.812	402	.793	486	.831
	fencing	883	.840	400	.860	483	.816
	holding a racket	890	.851	402	.858	488	.844
	bowling	888	.758	404	.727	484	.791
hand (bilateral)	holding a cue	867	.757	398	.824	469	.690
	baseball batting	847	.461	390	.397	457	.524
	holding a stick	812	.254	365	.175	447	.328
	boxing stance	819	.323	370	.417	449	.276
	golfing	832	.298	381	.232	451	.362
	target shooting	824	.599	383	.668	441	.544
	archery	861	.685	398	.737	463	.645
foot	kicking	834	.557	378	.437	456	.687
	long jump	814	.199	379	.104^b^	435	.292
	high jump	808	−.193	371	−.189	437	−.210
	skateboarding	773	.125	347	.094^ns^	426	.145^a^
rotation	rotating (vertical axis)	753	.044^ns^	329	−.027^ns^	424	.100^b^

*Note:* For the calculation of *r*_pb_ we excluded ‘no preference’ responses for the sport-specific tasks. The majority of relationships were significant with *p*<.001. To facilitate reading the table, only non-significant relationships or significant relationships with *p*-values larger than.001 are indicated as follows: ^ns^ *p>*.05, ^a^ *p*<.01, ^b^ *p*<.05. Please also note that we additionally clustered the participants into left- (LS <0) and right-handers (LS ≥0) and that we used these classifications to determine the relationship between handedness and sport-specific lateral preferences based on the calculation of phi coefficients Φ. In two calculation scenarios, once with inclusion and once with exclusion of ‘no preference’ responses, similar relationships as those reported above were found (see Table S3 for a summary of the results).

The above patterns in the relationships remain almost identical – irrespective of whether ‘no preference’ responses on the respective sport tasks are included or not – when participants are first classified into left- (LS <0) and right-handers (LS ≥0) [40] and then phi coefficients (Φ) are calculated as measure for the relationship between sport-specific lateral preferences and handedness (see Table S3). The data summarized in Table 3 shows that left-handers (males and females) demonstrated a right preference more often than right-handers demonstrated a left preference across the sport-specific tasks, except for foot preference in high jump. This pattern was particularly evident for unimanually controlled actions (e.g., throwing or holding a racket) for which right-handers clearly preferred the right hand.

**Table 3:** Left- and right-handers' lateral preferences for sport-specific tasks.

	Male				Female			
	Left-hander		Right-hander		Left-hander		Right-hander	
Task	L (%)	R (%)	L (%)	R (%)	L (%)	R (%)	L (%)	R (%)
throwing	32 (78.05)	9 (21.95)	4 (1.11)	357 (98.89)	32 (84.21)	6 (15.79)	3 (0.67)	445 (99.33)
fencing	35 (87.5)	5 (12.5)	2 (0.56)	358 (99.44)	28 (82.35)	6 (17.65)	3 (0.67)	446 (99.33)
holding a racket	33 (82.5)	7 (17.5)	2 (0.55)	360 (99.45)	32 (86.49)	5 (13.51)	2 (0.44)	449 (99.56)
bowling	27 (65.85)	14 (34.15)	4 (1.1)	359 (98.9)	29 (76.32)	9 (23.68)	4 (0.9)	442 (99.1)
holding a cue	35 (89.74)	4 (10.26)	9 (2.51)	350 (97.49)	29 (78.38)	8 (21.62)	17 (3.94)	415 (96.06)
baseball batting	23 (58.97)	16 (41.03)	36 (10.26)	315 (89.74)	27 (72.97)	10 (27.03)	37 (8.81)	383 (91.19)
holding a stick	19 (50)	19 (50)	78 (23.85)	249 (76.15)	28 (73.68)	10 (26.32)	96 (23.47)	313 (76.53)
boxing stance	30 (75)	10 (25)	56 (16.97)	274 (83.03)	28 (77.78)	8 (22.22)	143 (34.62)	270 (65.38)
golfing	20 (51.28)	19 (48.72)	58 (16.96)	284 (83.04)	27 (71.05)	11 (28.95)	82 (19.85)	331 (80.15)
target shooting	27 (72.97)	10 (27.03)	13 (3.76)	333 (96.24)	26 (70.27)	11 (29.73)	28 (6.93)	376 (93.07)
archery	30 (75)	10 (25)	9 (2.51)	349 (97.49)	30 (78.95)	8 (21.05)	20 (4.71)	405 (95.29)
kicking	22 (55)	18 (45)	27 (7.99)	311 (92.01)	27 (79.41)	7 (20.59)	14 (3.32)	408 (96.68)
long jump	19 (48.72)	20 (51.28)	130 (38.24)	210 (61.76)	29 (76.32)	9 (23.68)	111 (27.96)	286 (72.04)
high jump (foot)	18 (47.37)	20 (52.63)	252 (75.68)	81 (24.32)	12 (36.36)	21 (63.64)	274 (67.82)	130 (32.18)
skateboarding	24 (63.16)	14 (36.84)	159 (51.46)	150 (48.54)	21 (60)	14 (40)	158 (40.41)	233 (59.59)
rotating (vertical axis)	18 (50)	18 (50)	164 (55.97)	129 (44.03)	17 (58.62)	12 (41.38)	179 (45.32)	216 (54.68)

*Note*: For each sport-specific task, ‘no preference’ responses are excluded. Participants were classified as left- and right-handers based on their individual LS values (i.e., left-hander if LS <0 and right-hander if LS ≥0). ‘L’ and ‘R’ indicate left and right preference for a sport task.

## Discussion

This study furthers our understanding of sport-specific lateral preferences and their relationship with handedness in the normal population. While the proportion of left-handers in our sample was comparable to previously reported frequencies in males and females [4], [5], both sport-specific lateral preferences as well as the relationship between those preferences and handedness varied considerably depending on the task. These findings have several important implications which we will elaborate on in the following.

First, rather than indiscriminately referring to ‘handedness’ we suggest using more specific and differentiated terminology (e.g., fighting stance or orientation in boxing or MMA [34], shooting side or orientation in ice hockey [32]) when considering athletes' sport-specific lateral preferences. Doing so is necessary because of the inconsistent relationships found between sport-specific lateral preferences and handedness, particularly for bimanually controlled tasks. Athletes who demonstrate a left preference for tasks such as baseball batting or boxing are not necessarily left-handed as determined by handedness preference measures such as the EHI. We anticipate that usage of a more specific and differentiated terminology will reduce researchers' susceptibility to choose task-*un*related reference values (e.g., handedness for throwing) when testing, for example, if there is a higher incidence of left-*oriented* (‘southpaw’) fighters in boxing [12] or MMA [33]. Our call for more careful use of the term ‘handedness’ is also motivated by the term's typical use for describing someone's hand preference for *uni*manual, but not for bimanual, tasks [41], [42]. As a side note, we acknowledge that some researchers actually determined athletes' handedness through preference questionnaires and therefore the use of the terms left- and right-handedness in the respective works was justified (e.g., in boxing [43] and wrestling [44]). However, the problem is that those studies, in turn, missed to also report the athletes' lateral preferences for the respective domain-specific tasks (e.g., fighting stance) such that it remains unclear whether the differences discovered in fight records were actually due to mechanisms related to handedness, as proposed by the authors, and not to sport-specific lateral preferences in combination with sport-specific performance demands (for a critical review, e.g. see [14]).

Second, in conjunction with being more sensitive to the terminology, our findings highlight the need for task-specific normal population reference values when the aim is to identify if athletes with a left preference are overrepresented. This is a fundamental requirement particularly for sports where actions are bimanually controlled as in baseball or cricket batting, shooting in ice hockey, or fighting in boxing or MMA. Since left preferences were higher for such tasks compared to unimanual tasks (e.g., in our male participants the left preference for boxing was 2.4 times higher than the left preference for throwing), not controlling for task-specific reference values may result in biased results and conclusions. To exemplify, we re-analysed apparently enhanced frequencies of left-oriented performers in the elite domains of baseball (batting: 24.0%, *N* = 445, [12]), cricket (batting: 15.6%, *N* = 371, [12]), boxing (23.1%, *N* = 26, [12]) and MMA (20.4%, *N* = 245, [33]; 17.4%, *N* = 1468, [34]). Chi-square goodness-of-fit-tests revealed that, when task-specific reference values are considered (i.e., 14.46% and 21.23% left preference in males for batting and fight stance, respectively), a significant excess of left preference was only found for baseball batting (associated with a medium effect size, ω = .27, according to Cohen's conventions [45]). Thus, in contrast to predictions of the fighting hypothesis, left-oriented performers seem not overrepresented in samples taken from combat-like interactive sports such as boxing or MMA.

Third, albeit most relationships between sport-specific lateral preferences and handedness were statistically significant (*p*<.05; see Table 2), the fact that correlations were far from being ‘perfect’ challenges inferences as to a frequency-dependent maintenance of left-handedness in humans based on sports data. Not every athlete who demonstrates a left or right preference for sport-specific tasks must necessarily be left- or right-handed. A prominent example is the Spanish tennis professional Rafael Nadal, who plays tennis left-handed but is right-handed for most other tasks [46]. Therefore, sports data may be of limited suitability for testing evolutionary theories of handedness [12] or related models [10]. Similarly, the imperfect relationships between sport-specific lateral preferences and handedness cast doubt on alternative explanations like that an excess of performers with a left preference is due to handedness-dependent differences in neurological functioning (e.g., see [24], [43] for such argumentation). Generally, our findings demonstrate that it is difficult, if possible at all, to properly attribute increased frequencies or better fight records of athletes who have a sport-specific left preference to mechanisms associated with left-handedness [14], [25].

The data presented here might serve as reference values for future research on laterality effects in sports. In this regard, we would like to emphasize that we conservatively estimated the sport-specific left preferences by including all cases into the calculation of frequencies (i.e., ‘left’, ‘right’, and ‘no preference’ answers) instead of considering the number of ‘left’ and ‘right’ answers only. Thus, the percentages of sport-specific left preferences found here might even underestimate the “true” ratio of performers with a sport-specific left preference (raw frequencies are available in Table S2). Still, the frequencies identified here clearly show that sport-specific reference values are essential to adequately test observed frequencies of left preferences in sports for potential overrepresentation.

As pointed out earlier, our focus was on uni- or bimanually controlled actions in sports. With regard to non-manual actions, lateral preferences also varied considerably across the different sport tasks and relationships with handedness were moderate to low or even absent (see Table 2). Similar to previous work [27], the majority of participants preferred to use their right foot for kicking a ball or for jumping off in long jump. Also, most participants preferred their left foot for jumping off in high jump, which means that one has to approach the crossbar from the right side (please note that all participants were informed about this relationship in the questionnaire). The left foot preference in high jump appears remarkable in light of the predominance of right preferences for other tasks. A sound theoretical explanation for the occurrence of such left foot preference is missing up to now [47], [48], but speculating about it is beyond the scope of this paper. Furthermore, lateral preferences were least asymmetric for skateboarding and rotating along the vertical axis (see Figure 1). This pattern might be explained by task demands. More specifically, more than the other tasks investigated here, both hands and feet are substantially involved in the motor control of skateboarding or rotating along the vertical axis (e.g., in order to balance or to swing before spinning) and thus biased lateral preferences might not evolve to the extent known for other tasks such as throwing or baseball batting.

Finally, we would like to comment on two potential limitations of our work. First, we cannot completely rule out the possibility that some participants were not familiar with carrying out some of the sport tasks (e.g., holding a stick in ice hockey). Motor unfamiliarity might have affected those participants' indication of lateral preference for the respective items; although the direction of such potential biasing effect is unclear (i.e., over- or underestimation of left preferences). Previous work that assessed sport-specific lateral preferences in the normal population based on motor tests, however, found values similar to those reported here [27], [49]. Second, we determined our participants' handedness based on a preference questionnaire. While the EHI is one of the most widely used instruments to assess handedness and preference and performance measures of handedness appear highly correlated [42], inclusion of both preference and performance measures may still have resulted in better classification of left- and right-handedness [50]. We acknowledge this aspect as a potential limitation. At the same time, however, we suppose that even if we had included both types of handedness measurements into the protocol, similar varying relationships between sport-specific lateral preferences and handedness, as identified in the present work, would have emerged.

## Conclusions

Collectively, we showed that the frequency of the normal population's left preference depends on the sport task and that the relationship between sport-specific lateral preferences and handedness is highly variable. Task-specific reference values are mandatory when testing for an excess of ‘left-oriented’ athletes in sports. Also, we call for more careful use of the term ‘handedness’ within research on sport-specific lateral preferences for reasons discussed above. To help unravel the impact of handedness and task-specific lateral preference on high achievement in sports or related behavioural domains, future work is encouraged to include both preference and performance measures as well as to consider the performance demands associated with the domain of interest.

## Supporting Information

Table S1 Pictures used to facilitate understanding of left and right for sport-specific tasks.
(PDF)

Table S2 Lateral preferences for sport-specific tasks (raw frequencies).
(PDF)

Table S3 Relationship between sport-specific lateral preferences and handedness.
(PDF)
