# Supplementary material for: Left Preference for Sport Tasks Does Not Necessarily Indicate Left-Handedness: Sport-Specific Lateral Preferences, Relationship with Handedness and Implications for Laterality Research in Behavioural Sciences
Source: PLoS One. 2014 Aug 20;9(8):e105800. doi: 10.1371/journal.pone.0105800 (PMC4139391; doi:10.1371/journal.pone.0105800)
Supplement: Table S1 — Pictures used to facilitate understanding of left and right for sport-specific tasks. (PDF) [file pone.0105800.s001.pdf]

Table S1. Pictures used to facilitate understanding of left and right for sport-specific tasks.

Sport	Left	Right
baseball	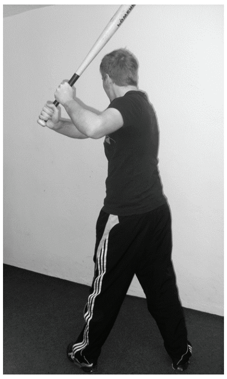	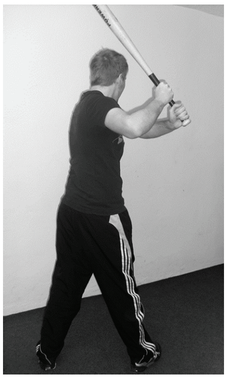
boxing	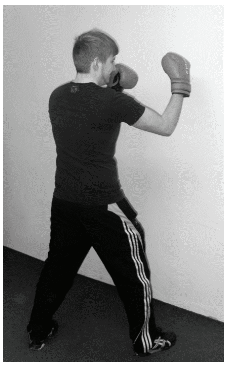	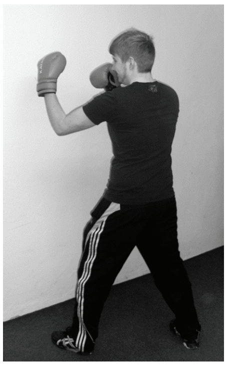
fencing	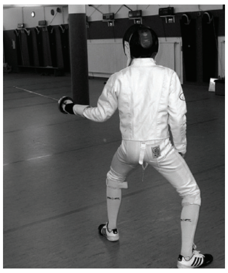	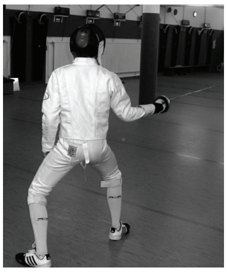
ice hockey	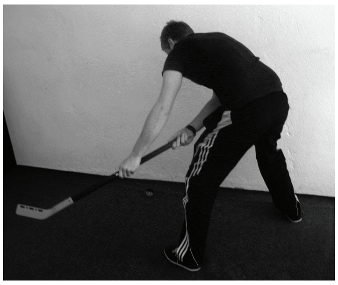	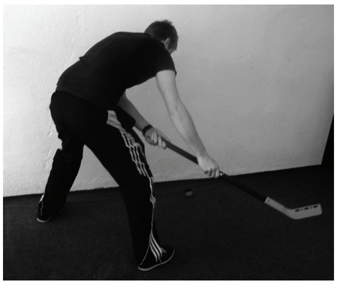

Table S1. ***(continued)***

Sport	Left	Right
racket sports<br>(e.g. table tennis,<br>tennis, badminton)	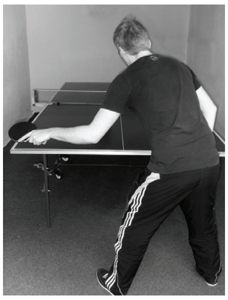	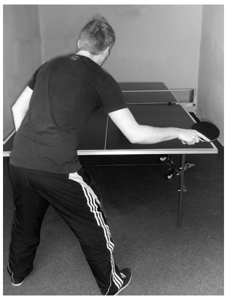
soccer	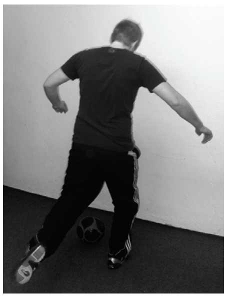	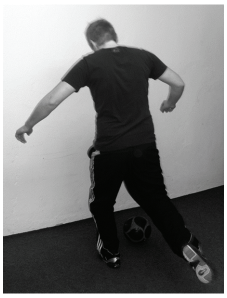
ninepin, tenpin<br>bowling	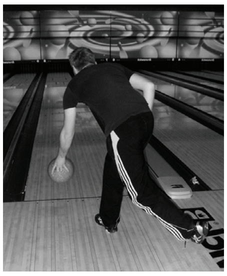	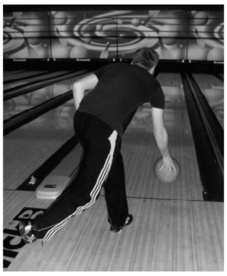
billiards	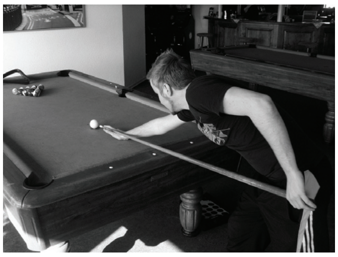	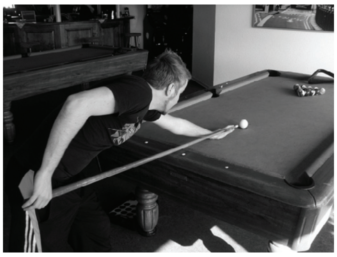
golf	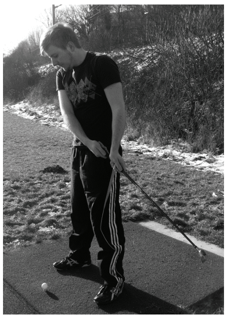	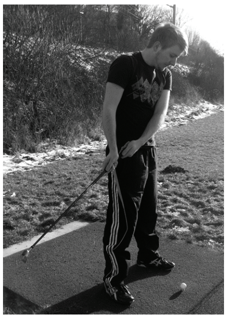

Table S1. ***(continued)***

Sport	Left	Right
target shooting	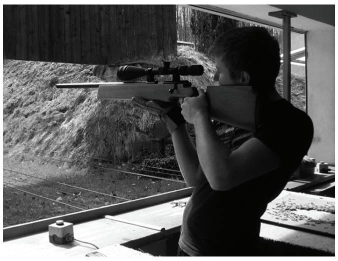	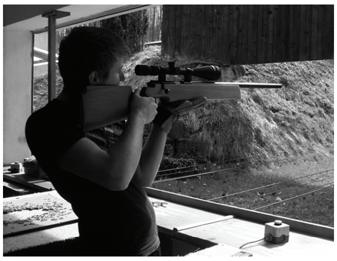
archery	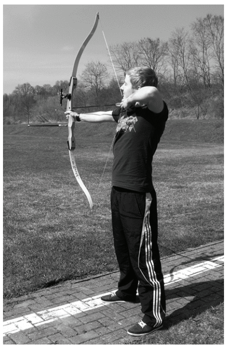	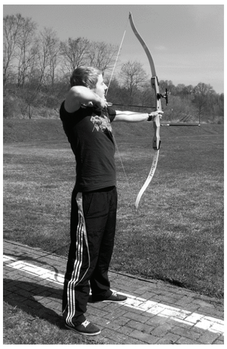
skateboarding, snowboarding	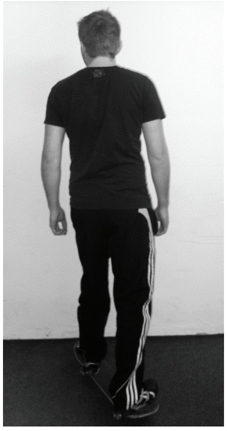	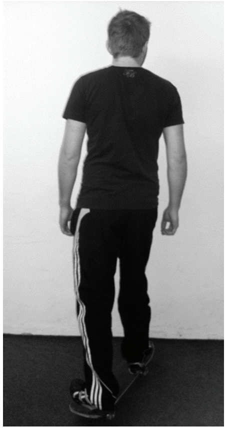
long jump	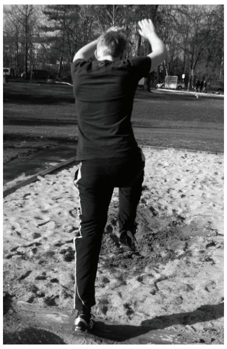	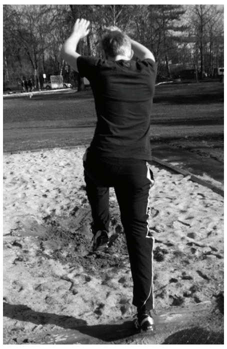

Table S1. ***(continued)***

Sport	Left	Right
high jump<br><br>(*Note*: Approaching from the left means jumping off with the right foot and vice versa. Participants were informed about this relationship.)	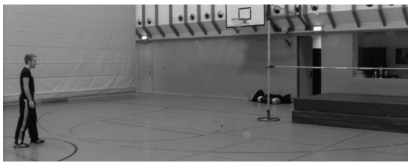	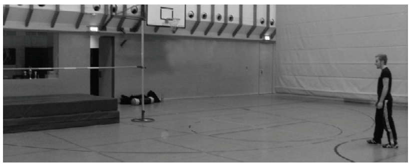
e.g. figure skating (rotation)	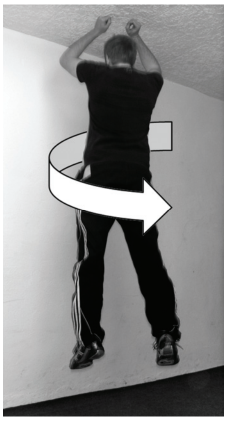	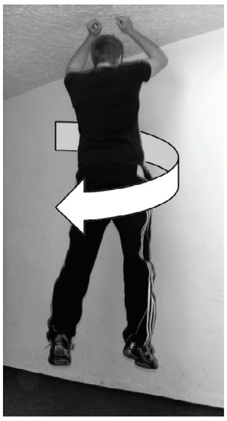
e.g. dart, team-handball (throwing)	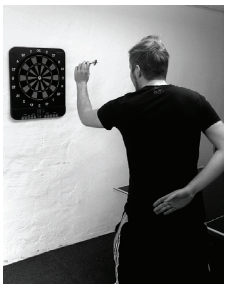	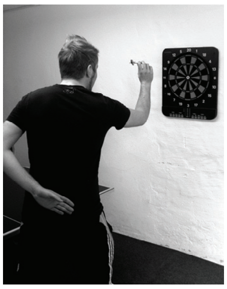
