# Supplementary material for: Left Preference for Sport Tasks Does Not Necessarily Indicate Left-Handedness: Sport-Specific Lateral Preferences, Relationship with Handedness and Implications for Laterality Research in Behavioural Sciences
Source: PLoS One. 2014 Aug 20;9(8):e105800. doi: 10.1371/journal.pone.0105800 (PMC4139391; doi:10.1371/journal.pone.0105800)
Supplement: Table S2 — Lateral preferences for sport-specific tasks (raw frequencies). (PDF) [file pone.0105800.s002.pdf]

Table S2. Lateral preferences for sport-specific tasks (raw frequencies).

Type of preference	Task (Sport)	Overall			Male			Female		
		Left	NP	Right	Left	NP	Right	Left	NP	Right
hand (unilateral)	throwing (e.g., darts, team-handball)	71	14	817	36	5	366	35	9	451
	holding a weapon (fencing)	68	19	815	37	7	363	31	12	452
	holding a racket (racket sports)	69	12	821	35	5	367	34	7	454
	bowling (ninepin, tenpin bowling)	64	13	824	31	3	373	33	10	451
hand (bilateral)	holding a cue (billiards)	90	33	777	44	8	354	46	25	423
	batting (baseball)	123	51	724	59	18	331	64	33	393
	holding a stick (ice hockey)	221	82	591	97	40	268	124	42	323
	stance (boxing)	257	77	562	86	35	284	171	42	278
	shooting orientation (golf)	187	65	645	78	27	303	109	38	342
	shooting orientation (target shooting)	94	67	730	40	19	343	54	48	387
	shooting orientation (archery)	89	36	772	39	8	359	50	28	413
foot	kicking a ball (soccer)	90	67	744	49	28	329	41	39	415
	jump off (long jump)	289	84	525	149	27	230	140	57	295
	jump off (high jump)	556	88	252	270	34	101	286	54	151
	front foot (skateboarding, snowboarding)	362	127	411	183	60	164	179	67	247
rotation	rotation along vertical axis (e.g., figure skating)	378	144	375	182	76	147	196	68	228

Note: 'NP' = no lateral preference
