# Supplementary material for: Left Preference for Sport Tasks Does Not Necessarily Indicate Left-Handedness: Sport-Specific Lateral Preferences, Relationship with Handedness and Implications for Laterality Research in Behavioural Sciences
Source: PLoS One. 2014 Aug 20;9(8):e105800. doi: 10.1371/journal.pone.0105800 (PMC4139391; doi:10.1371/journal.pone.0105800)
Supplement: Table S3 — Relationship between sport-specific lateral preferences and handedness. (PDF) [file pone.0105800.s003.pdf]

Table S3. Relationship between sport-specific lateral preferences and handedness.

Type of preference	Task	Inclusion of 'no preference'			Exclusion of 'no preference'		
		Overall	Male	Female	Overall	Male	Female
hand (unilateral)	throwing	.836	.816	.857	.841	.815	.868
	fencing	.855	.890	.824	.878	.900	.853
	holding a racket	.869	.861	.878	.882	.870	.895
	bowling	.765	.735	.795	.769	.735	.805
hand (bilateral)	holding a cue	.732	.811	.657	.750	.827	.675
	baseball batting	.445	.398	.491	.457	.408	.504
	holding a stick	.251	$.184^{a}$	.314	.250	.181	.313
	boxing stance	.304	.426	.232	.310	.427	.241
	golfing	.292	.252	.332	.295	.258	.332
	target shooting	.576	.646	.523	.595	.669	.536
	archery	.682	.724	.648	.690	.733	.657
foot	kicking	.536	.428	.656	.557	.431	.699
	long jump	.187	$.068^{ns}$	.299	.181	$.065^{ns}$	.292
	high jump	.168	.186	$.166^{a}$	-.178	-.193	-.175
	skateboarding	$.103^{a}$	$.099^{ns}$	$.107^{ns}$	$.096^{a}$	$.073^{ns}$	$.109^{b}$
rotation	rotating (vertical axis)	$.021^{ns}$	$.066^{ns}$	$.088^{ns}$	$.022^{ns}$	$-.038^{ns}$	$.067^{ns}$

Note: Here we categorized participants as left-handers (LS < 0) or right-handers (LS ≥ 0) and we used these classifications to determine the relationship between sport-specific lateral preferences and handedness (represented by phi coefficients, Φ). We calculated Φ twice to demonstrate that inclusion or exclusion of 'no preference' responses results in only marginally different relationships. The majority of relationships were significant with $p < .001$. To facilitate reading the table, only non-significant relationships or significant relationships with *p*-values larger than .001 are indicated as follows: $^{ns}\,p > .05$, $^{a}\,p < .01$, $^{b}\,p < .05$.

Also, please note that the above relationships for exclusion of 'no preference' are only marginally different to the point-biserial correlation coefficients representing the relationship between sport-specific lateral preferences and the raw LS values obtained from the EHI (see Table 2 in the main text).
